# Choices in vaccine trial design in epidemics of emerging infections

**DOI:** 10.1371/journal.pmed.1002632

**Published:** 2018-08-07

**Authors:** Rebecca Kahn, Annette Rid, Peter G. Smith, Nir Eyal, Marc Lipsitch

**Affiliations:** 1 Center for Communicable Disease Dynamics, Department of Epidemiology, Harvard T.H. Chan School of Public Health, Boston, Massachusetts, United States of America; 2 Department of Global Health & Social Medicine, King’s College London, London, United Kingdom; 3 MRC Tropical Epidemiology Group, London School of Hygiene & Tropical Medicine, London, United Kingdom; 4 Department of Global Health and Population, Harvard T.H. Chan School of Public Health, Boston, Massachusetts, United States of America; 5 Department of Immunology and Infectious Diseases, Harvard T.H. Chan School of Public Health, Boston, Massachusetts, United States of America

## Abstract

In a Policy Forum, Marc Lipsitch and colleagues discuss trial design issues in infectious disease outbreaks.

**Summary points**

- The 2014–2016 Ebola epidemic highlighted variations in the design of randomized trials to evaluate investigational vaccines in an emergency setting. Here, we summarize scientific, ethical, and feasibility considerations relevant to different trial designs.
- We focus on four fundamental choices in designing a trial of an experimental vaccine in the setting of an emerging infectious disease for which no proven vaccines exist: randomization unit, trial population, comparator intervention, and trial implementation. We also consider three ethical issues relevant to trial design: the social and scientific value of the trial, its risk–benefit profile, and the fairness of participant selection.
- We believe that individual rather than cluster randomization is better suited for estimating the direct protective effect of a vaccine, a measure of great intrinsic interest. Individual randomization should therefore be the default strategy for evaluating investigational vaccines during epidemics.
- Trial participants may be selected either from the general population or from a group at high risk of exposure to infection, depending on the characteristics of the infection together with statistical, fairness, and feasibility considerations.
- Use of a placebo control, rather than an active control or delayed intervention, is likely to maximize the social and scientific value of the trial because it facilitates double-blinding and removes concerns that the comparison intervention may affect the incidence of the disease under study.
- Starting the trial at approximately the same time for all participants should minimize the time required to obtain a result. Such a strategy will be facilitated when sufficient supplies of the investigational vaccine and control interventions (if any) are available at the start of a trial, when the geographic area for the trial is clearly identified (and anticipated to have continuing disease transmission throughout the trial), and when logistics permit rapid recruitment of the entire trial population. Otherwise, a stepped rollout may be necessary, in which recruitment to the trial is staggered over a period.

## Introduction

In outbreaks of emerging infectious diseases for which no proven efficacious vaccines exist but investigational vaccines have been developed, it is important both to rapidly test the investigational vaccines and, if effective, to deploy them. Following the 2014–2016 Ebola epidemic, the World Health Organization (WHO), the Coalition for Epidemic Preparedness Innovations, and other bodies committed to developing investigational vaccines for emerging infectious diseases [1,2]. They aim to evaluate them for immunogenicity and safety, so that promising candidates will be available for efficacy testing and possible deployment when an epidemic occurs.

In the Ebola epidemic, various strategies were used for the design of efficacy (i.e., Phase 3) trials for investigational vaccines [3]. Some investigators argued for individually randomized controlled trials (iRCTs), while others argued for forms of cluster-randomized controlled trials (cRCTs) [4,5]. Later in the epidemic, rapidly declining disease incidence required changes to some trial designs. Ideally, principles and protocols based on scientific, ethical, and feasibility considerations should be drawn up in advance of an epidemic, facilitating expediency and trust for rapid, early implementation once an epidemic occurs.

Here, we summarize key scientific, ethical, and feasibility considerations relevant to the design of Phase 3 vaccine trials in epidemic situations. Trial design choices are discussed, highlighting the benefits and drawbacks of each in given contexts.

## Scope

When designing and implementing randomized efficacy trials for investigational vaccines after safety and immunogenicity data have been collected (in Phase 1 and 2 trials), some key choices must be made. In the current regulatory system, randomized trials are considered the gold standard and, except in rare circumstances, have been required for vaccine licensure [6,7]. We restrict our scope to randomized trials of a single vaccine against an emerging infectious disease for which no effective vaccine exists. We assume that all participants, whether in the intervention or control group (if any), will have access to the best currently available other preventative measures (e.g., information on how to prevent infection).

We discuss four key elements of trial design: randomization unit, trial population, comparator intervention, and trial implementation. We weave into that discussion three important ethical considerations: the social and scientific value of the trial, its risk–benefit profile, and the fairness of participant selection [8,9]. These aspects are, in our views, key to trial design in these settings.

## Randomized vaccine trial design choices during epidemics

Table 1 summarizes the major designs that have been used or proposed for vaccine trials. Some have not been employed in epidemic settings.

**Table 1:** Possible trial designs to evaluate the efficacy of investigational vaccines during epidemics of emerging infectious diseases. The table does not provide an exhaustive list, and not all designs have been used for evaluating vaccines against emerging infectious diseases.

Number	Trial design type, with examples	Comparison	Population	Implementation
I. Individually randomized				
1	“Classic” individually randomized controlled trial* Ex: Pneumococcal vaccine, California [10] Rotavirus vaccine, Niger [11] PREVAIL Ebola vaccine, Liberia [12]	Placebo or other vaccine (“active control”)	General or high-risk	Parallel
2	Serodiscordant couples** Ex: Herpes simplex virus, type 2 [13]	Placebo	High-risk	Parallel
3	Individually randomized, comparison to delayed vaccination Ex: STRIVE Ebola vaccine, Sierra Leone, as performed [14]†	Delay (without a placebo)	High-risk	Stepped
4	Individually randomized controlled trial with deliberately stepped rollout Ex: Proposed for Ebola vaccines [15]	Placebo	General or high-risk	Stepped
II. Cluster randomized				
5	“Classic” parallel, cluster-randomized controlled trial Ex: Pneumococcal vaccine, Navajo [16]	Placebo or other vaccine (“active control”)	General	Parallel
6	Stepped-wedge design Ex: Hepatitis B vaccine, The Gambia [17] STRIVE Ebola vaccine, as initially proposed [18]	Delay (without a placebo)	General	Stepped
7	Ring-vaccination trial versus delayed vaccination Ex: Ebola ça Suffit Ebola vaccine, Guinea [19]†	Delay (without a placebo)	High-risk	Ring (stepped)

*Most common design used for vaccine efficacy trials. (A search on clinicaltrials.gov with filters "Interventional (or Clinical Trial)" for study type, "Phase 3" for study phase, and search term "vaccine" for intervention resulted in 1,251 trials, of which 989 were randomized. Out of a randomly selected 50 of these trials, all were individually randomized and 44 stated use of a parallel rollout.)

**Seronegative partner of a seropositive person is at high risk for exposure to infection and is randomized to vaccine or placebo.

^†^Choice of delayed vaccination comparison due to perceived challenges to the use of placebo in this setting.

Abbreviations: Ex., example; PREVAIL, Partnership for Research on Ebola Virus in Liberia; STRIVE, Sierra Leone Trial to Introduce a Vaccine Against Ebola.

### Randomization unit

Table 2 summarizes key features of iRCTs, in which vaccination is randomized between individuals in the same population, and cRCTs, in which groups of individuals are randomized. In iRCTs, individual-level protective effects are measured (also called direct effects, which can be thought of as the extent to which an individual’s risk of infection, or disease, would be reduced if he or she were the only person vaccinated). In cRCTs, population-level protective effects are measured (i.e., direct plus indirect effects, which take into account the reduction in the transmission of infection in a population if many are vaccinated) [20]. Either approach depends on the fact that, if the investigational vaccine is effective, the control group will be at greater risk of infection; the difference is simply how the membership of the control group is assigned [21].

**Table 2:** Key features of individually randomized and cluster-randomized trials.

Feature	Individually randomized trial	Cluster-randomized trial
Unit of analysis	Individuals randomized to the investigational vaccine or control arm. Ratio of participants in the investigational vaccine to control arm is typically 1:1 (most statistically efficient with fixed number of participants) but can be 2:1 or 3:1 (for increasing the safety database for the vaccine).	Clusters or groups of participants randomized to the investigational vaccine or control arm. Often, clusters are defined geographically (e.g., villages), but they can also be defined based on social contacts (e.g., Ebola ring vaccination trial) [19].
Differential in risk if vaccine proves effective	Between individuals who receive investigational vaccine and those who receive comparator	Between those in clusters randomized to receive vaccine and those in control clusters.*
Statistical efficiency	Greater statistical efficiency compared to cluster randomization [22]. All else equal, this leads to faster time to completion and earlier opportunity to administer a vaccine to control participants once it is judged effective [21].	Analysis incurs a statistical “penalty,” known as the design effect, to account for correlations in outcomes among members of the same cluster, leading to larger sample size requirements [23,24]. The design effect can be especially large when incidence is highly clustered in space and time [18,25–27].
Effects measured	Only measures direct protective effects [20].	Measures the combination of direct and indirect effects. Only in special circumstances can be designed and analyzed to elucidate the relative contributions of each effect.

*Note: If a “control” vaccine or placebo is used in the control clusters, then vaccine efficacy assessment can be focused on the comparison of disease incidence between those who actually received the vaccine in the vaccine clusters and those who actually received the control intervention in the control clusters. However, if no control vaccine or placebo is used in the control clusters (e.g., delayed vaccination), then the only unbiased comparison is between all those eligible to receive the vaccine in both types of cluster, including those who refused vaccination in the vaccine clusters (as this group cannot be separated out in the control clusters).

When testing an investigational vaccine during an epidemic, it is important to establish an efficacy estimate as rapidly as possible so that, if efficacious, the vaccine may still be deployed in the same epidemic. Also, in a declining epidemic, cases may become so rare that a trial is no longer feasible. iRCTs generally yield results more rapidly and that are easier to interpret than cRCTs and, in that respect, enhance the social and scientific value of a trial.

The fact that an iRCT measures the direct effect of a vaccine, whereas a cRCT measures the combined direct plus indirect effect, may favor either design. By including indirect effects, cRCTs provide a measure of protection closer to what might be obtained in widespread rollout of a vaccine. The combined effect may thus be of specific interest to decision-makers. However, indirect effects are more difficult to extrapolate to other settings than direct effects, the former depending on setting, population, network structure, and vaccine coverage [28–31]. A cRCT measures the protective effect that is highly relevant to the context in which the trial is conducted but may be less relevant at a later time in the same population, or in a different population. A cRCT’s direct and indirect effects cannot be easily separated, and it is possible that a vaccine’s performance would be different if another rollout strategy were used or in different settings within the same epidemic (e.g., urban versus rural) [32].

The direct vaccine effect, as measured in iRCTs, is likely to be less variable in different settings and, with assumptions about transmission dynamics, can be used to model indirect effects in different coverage and epidemic settings. Deriving an estimate of the direct effect from a cRCT is more complex and assumption dependent. Thus, we posit that the most valuable parameter to estimate in trials of unproven vaccines is the direct effect, as measured in an iRCT. Importantly, also, direct effects are generally the basis for regulatory decisions on the licensure of vaccines. For all these reasons, we believe that iRCTs should be the default design for evaluating investigational vaccines during epidemics.

Nonetheless, particular circumstances may weigh in favor of a cRCT. Recent work has shown that despite the larger sample size typically required in cRCTs compared to iRCTs because of the design effect (see glossary), in some settings, the difference in sample size may be modest, because the larger effect measured (indirect plus direct) in a cRCT partly offsets this effect [33]. Additionally, in some circumstances, an iRCT design may be logistically complex or may be unacceptable to the local population, which could threaten a trial’s successful completion—and thus its social and scientific value, essential to its justification [12,34]. In the Ebola epidemic, many considered a cRCT as the most feasible and acceptable design. However, with extensive community engagement, it was possible to launch an iRCT of an investigational Ebola vaccine in Liberia [12].

### Trial population

Trial participants may be selected either from the general population or from a group at high risk of exposure to infection.

When a vaccine is intended for widespread use in the general population, conducting the trial in the general population will enhance the generalizability of trial results. However, such trials will be feasible only if the incidence of the disease under study is high enough for a trial of manageable size. A vaccine trial conducted in persons at high risk of exposure, such as serodiscordant couples for a sexually transmitted infection [13] or healthcare workers for a disease transmitted by direct contact [14], is likely to reduce the required sample size and have greater statistical efficiency.

Efforts to enhance the risk–benefit profile of a trial may lead to performing a trial in a group that is especially likely to benefit if the investigational vaccine proves effective, such as those with occupational, familial, or household exposure to infection. Similarly, efforts to enhance a trial’s risk–benefit profile may favor excluding those who are most at risk from possible adverse effects of the investigational vaccine; these may include children, pregnant women or the fetuses they carry, or individuals with particular medical conditions, such as immune deficiencies. However, if such individuals would be in the eventual target population for a vaccination program, there are compelling arguments for including them in a trial.

Complexities ensue when these considerations conflict. Consider, for example, pregnant women and investigational vaccine trials against Zika virus infection. Concern about adverse effects on the fetus might argue for excluding all pregnant women. However, pregnant women and their fetuses are likely to benefit the most if an investigational Zika vaccine proves effective. Excluding them can make a trial’s risk–benefit profile significantly less favorable, by not collecting data on a key target population for the vaccine. Importantly, these considerations are also relevant for judging whether a trial’s participant selection is fair, inasmuch as compelling reasons are required for excluding entire population subgroups. A systematic precautionary approach has led to the previous exclusion of pregnant women from vaccine trials, even when they are an important target population for the vaccine [35,36]. The default should therefore be to include pregnant women and other so-called vulnerable groups in investigational vaccine trials during epidemics, provided that the risks of participation are judged acceptable [8,37].

For naturally immunizing infections, investigators sometimes restrict enrollment in a trial to those who have not previously been infected to ensure that trial participants are truly at risk of becoming infected; this is especially relevant when selecting individuals thought to be at high risk for infection. However, selecting participants who both have risk factors for infection and are uninfected at enrollment may be problematic. First, it means that all potential participants must be tested for evidence of prior infection. Second, individuals who have remained uninfected despite many opportunities for exposure may be more resistant to infection (or have lower-risk exposures) than is typical in the general population [38]. Serodiscordant couples, for example, may tend to be those who practice safer sex or for whom the infected partner is less infectious than in other couples. Likewise, healthcare workers who remain uninfected despite apparent intense exposure may be ones who practice excellent personal protection. In the early stages of the recent Ebola epidemic, health workers were identified as a group with particularly high incidence (and a good population for a vaccine trial), but when the high incidence was recognized, personal protective measures against infection were rapidly implemented, leading to a dramatic decline in their risk of infection. Failure to account for such factors may lead to overestimating the likely infection rate during the trial [38]. Moreover, if the vaccine is differentially effective in different populations, such as because of the intensity of exposure, then the effect estimate from such a trial may be different than it would be for the general population [39–41].

Targeting at-risk populations can promote fair participant selection, depending on how fairness is defined. When a population is at increased risk of infection because they are undertaking important work, such as burying the dead or caring for patients or family members, targeting this population serves fairness as reciprocity. However, focusing a trial on certain at-risk populations, such as frontline health workers, may invite charges of giving priority to those of relatively high social standing or undermining efforts to equalize access to the investigational vaccine. Ethical debate between these approaches to fairness is ongoing [42,43].

Practical or feasibility considerations may justify use of a high-risk group, for example, if there is a risk of obtaining an inconclusive result in a nontargeted trial, if the available supply of vaccine is limited so a smaller trial is necessary, or if there are substantial resource savings from conducting the study in a high-risk group.

### Comparator intervention

A trial may compare participants randomized to receive an investigational vaccine with those randomized to receive placebo, an active control (most commonly a proven effective vaccine against another infection), or delayed administration of the investigational vaccine.

The rationale for considering an active control (a vaccine against another disease) is to provide some benefit to the control group, which may enhance a trial’s risk–benefit profile, albeit not with respect to the disease under study. This is especially the case when the active control can be administered in such a way as to allow for double-blinding, as in the RTS,S/AS01 malaria vaccine trial, in which rabies and meningococcal C vaccines were used as active controls for infants and children, respectively [44]. However, use of an active control can complicate the safety assessment for the investigational vaccine, as only comparative safety between the two interventions will be measured [45]. Additionally, there may be a possibility that the control vaccine has an effect on the outcome measures against which the investigational vaccine is targeted. Active controls should therefore be considered but used judiciously.

Compared to both an active control and delayed intervention, use of a placebo control is likely to maximize a trial’s social and scientific value because of the opportunity for a double-blind design (neither investigators nor participants are aware of who has received the vaccine) and absence of concern that the placebo may affect the incidence of the disease under study in the control group. Use of a placebo control also facilitates assessment of adverse effects. Perhaps the strongest objection to using a placebo arises when the investigational vaccine is thought likely to be highly effective against a lethal disease (e.g., Ebola). Then, investigators may be thought to have a “duty to rescue” by providing the vaccine to all participants in a trial. While vaccines are a form of prophylaxis, not “rescue,” some may see a vaccine against a highly lethal disease as a form of rescue. However, we agree with Millum and Wendler that, in nearly all circumstances, the duty to rescue does not apply to clinical trials [46], when the intervention under investigation is unlicensed and of unproven efficacy and there is no alternative effective intervention. Placebo-controlled trials are typically conducted when the intervention under test is not licensed and is of unproven efficacy. A placebo control group is often scientifically necessary for assessing the investigational vaccine. In such circumstances, the loss for future populations from foregoing a placebo-controlled trial is too high [46]. The placebo control itself, typically a saline injection, introduces hardly any risk. We therefore believe a placebo control is typically the preferred comparator.

During an epidemic, political leaders or community representatives may have a strong preference for ensuring that all trial participants have access to the investigational vaccine, especially if prior evidence strongly suggests effectiveness (e.g., high immunogenicity and protection shown in animal studies), the disease is serious, the disease burden is high, and available preventative or therapeutic measures are limited. If this preference makes use of a placebo control unfeasible, delayed administration may be an alternative, provided the social and scientific value of the study remains intact [47]. When the investigational vaccine is expected to be effective, delayed administration can enhance the trial’s risk–benefit profile compared to a placebo control. The major disadvantage is that individuals clearly know if they are in the vaccine or control group, and this may lead to differential behavior changes, affecting their risk of disease independently of any protective effect of the vaccine. In cRCTs, another disadvantage of assigning control clusters to delayed administration (rather than active or placebo control) is that it may be impossible to identify those in the control clusters who would have been vaccinated had they been offered the vaccine. As acceptors and refusers may be at differential risk of disease independently of vaccination, the only possible unbiased comparison is disease rates among all those in the vaccine clusters compared to those in the control clusters—an intention-to-treat analysis. If substantial numbers refuse vaccination, the vaccine’s effect may be underestimated. A final disadvantage of delayed administration is that it may compromise assessment of the longer-term efficacy of the vaccine. This concern may be moot, however, if the plan is to offer the vaccine to all participants after the trial [47], in which case such an evaluation would be impossible, anyway, regardless of the comparator used in the trial.

### Trial implementation

In the simplest experimental trial design, known as a “parallel” design, all participants are enrolled into the trial at about the same time and followed for the same period of time. However, sometimes it is not feasible to enroll all participants in a short period and a “stepped” rollout is used, in which entry to the trial is phased over time. While some degree of stepped rollout occurs in almost all trials (not all participants can be vaccinated on the same day), stepped rollout has been used deliberately in cRCTs. In a so-called “stepped-wedge” cRCT, the order of introduction of the vaccine to the various clusters is randomized, and disease incidence is then compared in successive time intervals between those clusters in which the intervention has already been rolled out and those in which it has not yet been rolled out [17]. By the end of the study, all clusters will have received the investigational vaccine.

Deliberate rollout of the investigational vaccine over a period of time may be implemented because of limited supply of the investigational vaccine or limited capacity to implement the intervention in many locations simultaneously [48]. A stepped rollout can permit prompt commencement of the trial, without waiting until rollout everywhere is feasible. Low and variable disease incidence may also make parallel rollout unfeasible, because the trial can gain sufficient power only by targeting at-risk populations and randomizing individuals or clusters in the vicinity of known or predicted cases [15,19,49]. Such trials, following cases in real time, are necessarily rolled out in a stepped fashion.

Stepped rollout does not have this same advantage if the order of rollout is fixed in advance, as, for example, in a classic stepped-wedge cRCT design, in which clusters (e.g., villages) are offered an investigational vaccine in an order predetermined by randomization. This design was considered during the Ebola epidemic when supplies of investigational vaccines were limited. However, in this case the design was poorly suited to a setting with unpredictable and spatially variable incidence, because much variation would occur between clusters because of factors other than the vaccine, inflating the design effect and reducing the trial’s power. The solution proposed was an iRCT with parallel rollout, which would have greater power than a stepped-wedge design and thus greater social and scientific value [18]. However, an iRCT with a stepped rollout to areas of likely high incidence as supplies become available was also proposed, combining the advantages of stepped rollout with the greater power of an iRCT [15,21].

The Ebola experience shows that conditions favoring stepped rollout can readily occur in an emergency setting. This was an important part of the justification for the ring vaccination design of the “Ebola ça Suffit!” trial. Notably, some conditions (limited supplies and logistics) were particularly acute at the start of the epidemic, while others (variable disease incidence) were particularly pressing at the end. When sufficient supplies of the investigational vaccine and any control interventions are available at the trial’s start; when the trial’s geographic area is clearly identified and anticipated to have continuing transmission throughout the trial; and when logistics permit large-scale rollout simultaneously to the entire trial population, starting the trial at a similar time in all participants will minimize the time required to obtain a result. When these criteria are met, a parallel rollout should be used.

## Conclusion

We argue that individually randomized trials with a placebo control should be the default strategy for evaluating investigational vaccines during epidemics. Placebo-controlled trials typically maximize the social and scientific value of the trial, and objections to using placebo, such as a duty to “rescue” individual participants—with an unlicensed investigational vaccine candidate of unproven efficacy—are rarely persuasive. Depending on the pathogen as well as statistical, fairness, and feasibility considerations, trial participants may be selected either from the general population or from a group at high risk of exposure to infection. Starting the trial at approximately the same time for all participants, in a parallel rollout, will minimize the time required to obtain a result and maximize social and scientific value. If resources are limited and/or incidence is spatiotemporally variable, a stepped rollout may be necessary, in which recruitment to the trial is staggered over time.

We have not discussed all aspects of vaccine trial design during epidemics of emerging infectious diseases. However, through a discussion of four fundamental choices and three ethical considerations, we hope to have highlighted the range of options that should be considered during future epidemics. A closely overlapping set of decisions has recently been incorporated into an online interactive decision tool as part of the WHO Research and Development Blueprint for Action to Prevent Epidemics (the InterVax-Tool: http://vaxeval.com/) [50].

In order to further prepare in advance for epidemics, future work should characterize choices for specific pathogen characteristics and contexts. Mathematical modeling and simulation are useful tools for addressing choices beyond the four discussed here, e.g., sample size, trial location, duration, or end point [33,50–52]. Rigorous ethical analysis, as well as inclusive and transparent debate by different stakeholders, would help to illuminate the underlying moral questions. Ethical considerations not discussed here, such as informed consent and community acceptance, may also tip the balance between several possible designs. Both scientific and ethical questions would be best debated in advance of future epidemics.

**Glossary**

- **Blinded design**: In a blinded design, the trial’s participants do not know whether they are receiving the investigational vaccine or the control. In a double-blinded design, neither the trial’s participants nor the investigators know who is receiving the investigational vaccine or the control. In non-blinded trials, bias can arise in intervention allocation if, for example, the investigators knowingly put the more or less vulnerable participants in the investigational vaccine arm, or if participants change their behavior if they know they did or did not receive the investigational vaccine.
- **Design effect**: A statistical “penalty” incurred in the analysis of cRCTs to account for correlations in outcomes among members of the same cluster, leading to larger sample size requirements [23,24].
- **Direct effects**: The extent by which the risk of disease is reduced when an individual is exposed to the infectious agent. The direct effect of a vaccine is mainly a characteristic of the vaccine itself and how it interacts with individuals rather than of the way the vaccine is deployed in a particular population. While direct vaccine effects are not always generalizable [29,40,41,53], they are often assumed to be more easily extrapolated to other settings because direct effects are measured at the individual level and not typically thought to depend much on the patterns of exposure and transmission in the population.
- **Effective/efficacy**: We use effective and efficacy in this paper as shorthand for protection against becoming infected without prejudging various aspects of this protection, which are sometimes called efficacy and effectiveness.
- **Indirect effects**: Indirect effects occur when the number of persons vaccinated in a community reduces the overall transmission rate of the infection in the community. Sometimes called herd effects, indirect effects benefit both vaccinated and unvaccinated individuals, and the size of the effect will depend both on the level of the direct effect and the proportion of persons vaccinated in the population [20]. The indirect effects of a vaccine depend not only on the direct effects on vaccinated individuals but on such factors as the incidence rate of the disease, the level of pre-existing immunity in the population, the contact network structure, the coverage of the vaccine, and the phase of the epidemic (growing or declining), among others. Each of these factors will vary across populations that may consider using the investigational vaccine if it proves effective, and some of them (e.g., epidemic phase, immunity) may vary over short time periods within the same population.

## Abbreviations

cRCT: cluster-randomized controlled trial
iRCT: individually randomized controlled trial
PREVAIL: Partnership for Research on Ebola Virus in Liberia
STRIVE: Sierra Leone Trial to Introduce a Vaccine Against Ebola
WHO: World Health Organization
